# Face perception and processing in early infancy: inborn predispositions and developmental changes

**DOI:** 10.3389/fpsyg.2015.00969

**Published:** 2015-07-09

**Authors:** Francesca Simion, Elisa Di Giorgio

**Affiliations:** ^1^Department of Developmental and Social Psychology, University of Padova, Padova, Italy; ^2^Center for Cognitive Neuroscience, University of Padova, Padova, Italy; ^3^CIMeC, Center for Mind/Brain Sciences, University of Trento, Trento, Italy

**Keywords:** face perception, face processing, early infancy, perceptual narrowing, visual experience

## Abstract

From birth it is critical for our survival to identify social agents and conspecifics. Among others stimuli, faces provide the required information. The present paper will review the mechanisms subserving *face detection* and *face recognition*, respectively, over development. In addition, the emergence of the functional and neural specialization for face processing as an experience-dependent process will be documented. Overall, the present work highlights the importance of both inborn predispositions and the exposure to certain experiences, shortly after birth, to drive the system to become functionally specialized to process faces in the first months of life.

## Introduction

The ability to detect and to discriminate social beings from inanimate objects is of paramount importance to survive. Among other social cues in the environment, faces are probably the most important to us as humans, since they convey relevant social information, such as identity, age, gender, emotions. Humans are expert in processing faces, and evidence from behavioral, brain lesion, and neuroimaging studies suggests that, in adults, face processing involves specific face processing strategies (i.e., *functional specialization*, Farah et al., 2000) carried out by dedicated brain areas (i.e., structural or neural specialization, Allison et al., 2000; Kanwisher, 2000, 2010). Together, these findings support the hypothesis that the adult brain is equipped with a neural circuitry specialized for preferentially processing faces (Haxby et al., 2002; Haxby and Gobbini, 2011).

As regard with *neural specialization*, according to the models proposed by Haxby (Haxby et al., 2000; Haxby and Gobbini, 2011), face processing in humans recruits a complex and distributed neural system comprised of multiple regions. This system is formed by a “core system” and an “extended system” that work in concert. The core system comprises three functionally distinct regions of extrastriate cortex in both hemispheres: the inferior occipital region, which contributes to early stage of face perception, provides input both to the lateral fusiform gyrus (including the fusiform face area, FFA) for the processing of invariant characteristics of faces, and to the superior temporal sulcus (STS) for the processing of changeable aspects. The authors suggested that, to analyze all the information embedded in a face, it is necessary to postulate reciprocal interconnections between the core system and the extended system, which comprises brain structures responsible for other cognitive functions (i.e., frontal eye fields, intra-parietal sulcus, amygdala). This distributed neural network maps, at a functional level, the cognitive model of face processing proposed by Bruce and Young (1986). This model suggested that face processing is divided into two different processes: *face detection*, which implies the capacity to perceive that a certain visual stimulus is a face, and *face recognition*, that is the capacity to recognize whether a face is familiar (e.g., already seen before) or not and, successively, to identify the identity of a specific face.

As regard with *functional specialization*, evidence from adults’ studies has shown that faces are special and are processed in a more holistic or configural way than objects (Tanaka and Farah, 1993; Farah et al., 1998; but see also Robbins and McKone, 2007). To recognize faces, we employ different strategies that require to process different information: the shape of single facial features (i.e., featural information), the space among inner facial features (i.e., second-order configural information) and the global structure of the face (i.e., holistic information; Maurer et al., 2002; Piepers and Robbins, 2012). The inversion effect, the composite face effect and the part-whole effect corroborate the notion of specific strategies in face processing as compared to the strategies adopted to process other objects.

The “*face inversion effect*” (FIE) refers to impairments in the configural information processing from inverted faces compared to other classes of objects (Rossion and Gauthier, 2002, for a review, Yin, 1969). This effect has been considered as the most critical marker for configural face processing in adults, even if some authors hypothesize that the inversion effect is a marker for the adult ability to process and recognize both the configual and featural information embedded in faces. Indeed, some evidence has been grounded that inverting a face affects the capacity to process featural as well as configural information (Rhodes et al., 1993; Malcolm et al., 2004; Riesenhuber et al., 2004; Yovel and Kanwisher, 2004).

The “*composite face effect*” refers to the phenomenon by which the recognition of the two halves of different faces is more difficult when they are horizontally aligned compared to when they are misaligned. In the aligned condition only, the two halves create the illusion of a novel face and therefore adults process it holistically. For this reason, this effect is considered a marker for holistic face processing (Young et al., 1987; Hole, 1994; Rossion, 2013), as well as “*the part-whole effect*” where subjects demonstrate to be more accurate in recognizing the identity of a face feature when it is embedded in the whole face (Maurer et al., 2002).

At first glance, the existence of specific brain areas and of specific strategies for face processing fits well with the idea that they are products of natural selection due to their survival value. For this reason, they are hypothesized to be domain-specific and likely innate (McKone et al., 2006; Wilmer et al., 2010; Zhu et al., 2010). Alternatively, as the experience-dependent hypothesis suggests, the existence of regions specialized for face processing might be the result of the extensive experience with this category of visual stimuli during lifetime (Gauthier et al., 1999; Tarr and Gauthier, 2000; Bukach et al., 2006). Within this open debate, a developmental approach becomes critical to answer the question about the origin of face specialization and whether the functional and structural specialization for face processing, found in adults, is present from birth or is the product of a progressive specialization attributable to visual experience.

Some data seem to contradict the hypothesis of a late and progressive specialization for face processing, because the available evidence, coming from both humans and non-humans, demonstrate early predispositions to orient to faces and renders the hypothesis of a late specialization uncertain. In effect, 2 day-old newborns, despite their lack of experience, orient preferentially toward face or face-like configurations rather than to other, equally complex, non-face stimuli (Goren et al., 1975; Morton and Johnson, 1991; Valenza et al., 1996; Macchi Cassia et al., 2004). Newly hatched chicks attend at patterns similar to the head region of their caregivers (Rosa Salva et al., 2011). Similarly, newborn monkeys, without any visual experience with faces, manifest a preference for faces as compared to objects (Sugita, 2008).

In light of the above evidence in the present paper empirical findings will be reviewed on the mechanisms that subserve face preference (i.e., *face detection*) and face recognition at birth and on the progressive structural and functional specialization of the system to faces during development.

### General or Specific Mechanisms Underlying Face Preference at Birth?

Different interpretations were proposed to account for human newborns’ face preference, in terms of both domain-specific or of domain-general mechanisms underlying it.

Johnson and Morton (1991) proposed a two-process model of face processing, more recently updated (Johnson, 2005; Johnson et al., 2015), which hypothesizes that newborns possess a first face specific subcortical mechanism, named *Conspec*, to detect faces, selectively tuned to the geometry of a face, and a second, domain-relevant cortical mechanism, named *Conlearn*, that comes to specialize in face recognition. The subcortical mechanism guides the cortical one to acquire information about faces. In this model, face detection at birth is due to Conspec, the face-sensitive mechanism adapted for perceiving conspecifics (Johnson and Morton, 1991), later defined as a subcortical low-spatial frequency (LSF) face specific detector, provided by evolutionary pressure active throughout the life span (Tomalski et al., 2009). This subcortical detector would guide the cortical areas that, later during development, will constitute the face network. Specialization of the face cortical circuits would emerge by the interaction of the subcortical mechanism that biases infants’ visual attention toward faces and the experience with faces. Importantly, a recent neuroimaging study with newborns corroborated the idea that also the visual cortex contributes in part to the development of the face processing system starting from birth (Farroni et al., 2013), supporting the hypothesis that both subcortical and cortical mechanisms are present at birth (Acerra et al., 2002) and interact (Nakano and Nakatani, 2014). According to this model, the domain-specific mechanism supporting face detection allow newborns to orient to faces and, at the same time, biases the cortical circuits that, progressively will become specialized for face processing.

The existence of a mechanism specifically devoted to detect faces in the environment has been questioned by an alternative view (Simion et al., 2001, 2003, 2006; Turati, 2004) that proposed to explain newborns’ preferences as due to domain-general attentional biases toward some structural properties present in a face as well as in other non-face like objects. According to this hypothesis, these general attentional biases are not specifically adapted for detecting faces, and likely derive from the functional properties of the immature newborn’s visual system and they are applied in the same manner at faces and non-face stimuli. Indeed, they are *domain-relevant* because allow newborns to successfully detect and identify faces when embedded among other non-facelike stimuli (Simion et al., 2001). This view is consistent with the notion that newborns’ visual system is immature and is sensitive not only to a certain range of spatial frequency, as described by the contrast sensitivity function (CSF; see Acerra et al., 2002 for a computational model), but also to other structural higher-level Gestalt-like properties, as demonstrated by newborns’ preference for horizontal versus vertical stripes (Farroni et al., 2000). From this point of view, faces would be preferred because they are a collection of perceptual structural properties that attract newborns’ attention. In effect, faces are symmetric along the vertical axis, contain areas of high contrast (i.e., the eyes) and have more elements in their upper part displaced congruently with the external outline. In addition, faces are three-dimensional, move and, importantly, manifest a behavior contingent upon the baby’s activities. All these characteristics are present simultaneously in faces and render them probably the most interesting stimulus experienced by newborns.

**FIGURE 1:**
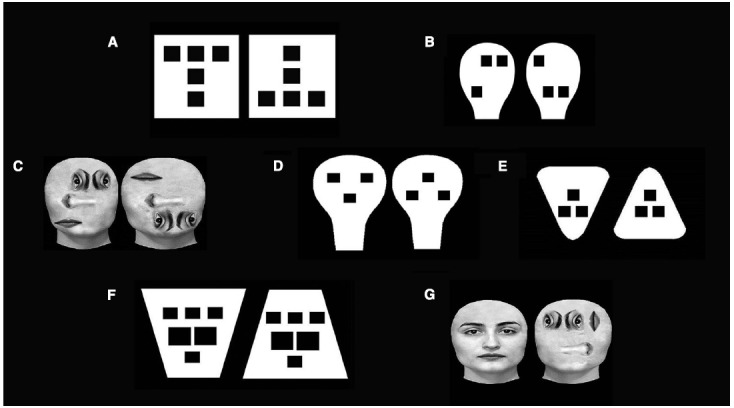
**Examples of stimuli employed by to test the role of general structural properties in face preference. (A,B)** stimuli used to test up-down asymmetry (Simion et al., 2002; Turati et al., 2002); **(C)** real faces employed to test up-down asymmetry (Macchi Cassia et al., 2004); **(D–F)** stimuli used to test congruency (Macchi Cassia et al., 2008); **(G)** real faces employed to test up-down asymmetry and congruency (Macchi Cassia et al., 2004).

Data from our lab showed that at least two non-specific structural properties can elicit newborns’ preference both for faces (Turati et al., 2002; Macchi Cassia et al., 2004) and geometric configurations (Macchi Cassia et al., 2002, 2008; Simion et al., 2002). A first property, termed *up-down asymmetry* (or top-heaviness), “*is defined by the presence of higher stimulus density in the upper than in the lower part of the configuration*” (Simion et al., 2002; Turati et al., 2002; Macchi Cassia et al., 2004). In effect, newborns preferred geometrical stimuli with more elements in the upper part when contrasted with the upside-down version of them (Simion et al., 2002 see Figure 1A). The same results were replicated with face-like stimuli (Turati et al., 2002, see Figure 1B) and with real faces (Macchi Cassia et al., 2004, see Figure 1C) in which the geometry of the face was disrupted. These data suggest that this up-down asymmetry, if compared with the face geometry or face structure, is the critical factor in eliciting newborns’ preference. This visual preference for configurations with more elements in the upper part may originate from an upper-field advantage in visual sensitivity that renders those configurations more easily detectable (Simion et al., 2002). This sensitivity is attributed to the fact that a major role in visual exploration of the upper visual field is played by the superior colliculus (Sprague et al., 1973), which is thought to affect preeminently newborns’ visual behavior (Atkinson et al., 1992).

The second non-specific property is the *congruency* –“*i.e., presence of a congruent or corresponding relationship between the shape and orientation of the contour and the spatial disposition of the inner features*” (Macchi Cassia et al., 2008). Faces are congruent because they display a greater number of features (the eyes) in the wider, upper portion of the face outline and only one feature (the mouth) in the narrower part (see Figure 1D). Evidence revealed that when congruent and non-congruent non-face geometrical configurations were compared (using both triangles and trapezoids, see Figures 1E,F), newborns looked longer at the congruent pattern (Macchi Cassia et al., 2008). There are several reasons why newborns preferred congruent configurations compared to non-congruent ones. First, in line with some Gestalt-like principles, congruent visual stimuli are easily processed by the visual system from birth because they fit well with the figural simplicity and regularity criteria (Palmer, 1991). Second, newborns perceive and detect configural information embedded in hierarchical stimuli better than featural information (Macchi Cassia et al., 2002; Simion and Leo, 2010).

Overall, since newborns’ visual behavior was affected by the up-down arrangement of the inner features and by congruency, independently of whether such arrangement was or not face-like, these findings support the hypothesis of the existence of general non-face specific attentional biases toward structural properties of the stimuli. Their presence at birth seems sufficient to cause the human face to be a frequent focus of newborns’ visual attention, allowing the gradual development of a face representation and of a face processing system.

Intriguingly, top-heaviness and congruency are two important structural properties that play a role in shaping the response of adults’ face sensitive areas, highlighting the findings obtained with newborns. An fMRI study showed that adults’ face cortical areas (e.g., FFA) are tuned for patterns with more elements in the upper part, even if these patterns were not perceived as face-like stimuli (Caldara et al., 2006). This result corroborates the idea that up-down asymmetry is crucial in eliciting face preference not only at birth, but also in adulthood. In addition, the same structural properties (i.e., top-heaviness and congruency) modulate the latency and the amplitude of early face-sensitive ERP components in adults (e.g., P1 and N170). Crucially, the violation of both these structural properties modulates ERP components more than the violation of each property alone, demonstrating that they produce an additive effect in face preference (Macchi Cassia et al., 2006).

The existence of general attentional biases toward perceptual and structural properties to explain face preference is in line with a recent theoretical Binocular Correlation Model (i.e., BCM) that proposes to explain the neonatal face bias as a result of a visual filtering mechanism, related to the limited binocular integration possessed by newborns (Wilkinson et al., 2014). In other words, face-like and non-face-like stimuli were presented in the center of a robot’s visual field and the salience value was recorded. A binocular model was compared to a monocular model. Results obtained from the binocular model resembled the face preference found in newborns. Although the BCM was able to generate a face preference, the authors suggest that “ *it is not based on an innate internal representation of facial structure. It relies on generic binocular circuitry, not a specialist module*” (Wilkinson et al., 2014). In addition, the same model can explain both face preference at birth and other visual preferences that have nothing to do with faces. For example, the BCM model suggests that horizontally oriented patterns are preferred because they generate more binocular correlation than vertical ones. The same hypothesis is true for stimuli with more elements in the upper part. Although further empirical studies are needed to confirm these hypotheses, it seems that the BCM model is a promising computational model to investigate the mechanisms underlying face preference at birth.

The hypothesis of the existence of general biases to explain face preference at birth has been undermined by a study that highlighted how the contrast polarity of the stimuli is determinant to induce such a preference (Farroni et al., 2005). The rationale was that, if the up-down asymmetry is crucial to determine face preference, then the contrast polarity of the elements should not interfere (i.e., face-sensitive view, see Johnson et al., 2015, for a discussion). Results demonstrate that in the negative polarity condition the preference for upright face-like stimuli disappears (see Rosa Salva et al., 2012), for a similar result in newly-hatched chicks. Consistent with that, the authors proposed that the newborns’ visual system has been shaped, by natural selection, to prefer faces in the environment under natural lighting illumination conditions, which are from above rather than from below.

Unfortunately, the absence of significant results (i.e. null results) under the negative contrast polarity condition between upright and inverted face-like patterns cannot be considered conclusive, because alternative explanations are possible. First, a large number of stimulus variables, as the sensory hypothesis proposed, can affect newborns’ preferences. In particular, at birth, the attractiveness of a pattern is affected by the amplitude spectra (i.e., contrast, luminosity, spatial frequency) as well as by the phase spectra (i.e., structural properties; Slater et al., 1985). The reversal of contrast polarity can be described, in the spatial frequencies domain, as 180°shifts in the phase angles of all spatial frequencies and this shift could interfere with newborns’ preferences for faces (Mondloch et al., 1999) and for both faces and objects in 6-week-old infants (Dannemiller and Stephens, 1988). Second, the phase spectra of certain patterns cannot be arbitrarily shifted without destroying the discriminability of the pattern (Kemp et al., 1996) since a change in polarity might affect the process of figure-ground segregation: black regions are more often perceived as figures. Future studies, which either verify if the contrast polarity effect is limited to face-like patterns or if the change in polarity decreases the discriminability of stimuli other than faces, are required to test the role of contrast polarity in determining newborns’ preferences. Finally, a mechanism underlying face preference which is more face-related than previously supposed, cannot explain the data demonstrating that an upright stimulus with three blobs randomly located in the upper part is always preferred over a face-like pattern (Turati et al., 2002) and that a scrambled face with more elements in the upper part is always preferred to a real face (Macchi Cassia et al., 2004, see Figure 1G).

Consequently, if one takes into account all these considerations, it clearly appears that we are still with two possible interpretations of face preferences at birth and that we are far from a conclusive answer to the question about general domain relevant attentional biases or a specific LSF face detector to explain face preference at birth. What we know, for sure, is that these attentional biases cannot explain face preferences later during development, because 3-month-old infants prefer to look at faces even when they were contrasted with scrambled face configurations with more elements in the upper part (Turati et al., 2002), corroborating the idea that 3 months of visual experience are sufficient to change and tune the face representation.

### Developmental Changes in Face Representation

Behavioral evidence supports the idea that face representation changes over development and that experience allows infants to build up a specific representation of experienced faces and to categorize faces within a face space (Valentine, 1991; Valentine et al., 2015).

The face space is “*defined as a multidimensional space, in which each individual face is coded as a point in a continuum where the average face lies at the center of the space*” (Valentine, 1991). This face space narrows over time as a function of experience, so that infants become expert in processing the most experienced faces as proposed by the *perceptual narrowing* view (Nelson, 2001, 2003). According to this view, infants begin life with general mechanisms dedicated to processing faces as well as other stimuli and subsequently become “tuned” to the experienced human faces, as a direct consequence of the exposure to this kind of visual stimuli present in the species-specific environment during the first months (Scott et al., 2007).

Data from both human and non-human infants corroborate the hypothesis of the existence of a broad face perception system at birth. A large proportion of the literature on face-perception at birth in both non-humans (Sugita, 2008) and humans (Kelly et al., 2005; Quinn et al., 2008) reveals clear evidence of a basic, coarsely tuned face-perception system in primates as well as in humans that becomes tuned to the experienced faces. For example, newborns do not show any visual preference for faces from their own or other ethnic groups (Kelly et al., 2005), in contrast this effect is present few months later (Kelly et al., 2005; Anzures et al., 2013). In the same vein, newborns do not respond differentially to the gender of the faces (Quinn et al., 2008), but 3 months of experience are enough to elicit it (Quinn et al., 2002). Furthermore, newborns do not prefer a human face when contrasted with a non-human monkey face equated for all the low-level perceptual properties (i.e., high contrast areas or spatial frequencies; Di Giorgio et al., 2012; but see Heron-Delaney et al., 2011). This preference appears 3 months later (Heron-Delaney et al., 2011; Di Giorgio et al., 2013; Dupierrix et al., 2014).

Interestingly, Di Giorgio et al. (2012) bring into question also the role of the eyes in triggering newborns attention toward faces, since the contrast between the sclera and the iris, which is present in human eyes but not in the non-human ones, does not determine any preference. Recently, Dupierrix et al. (2014) confirmed this result. Newborns that were simultaneously presented with a pair of non-human primate faces differing only for the eyes do not manifest any preference between a face with original non-human primate eyes and the same face where the eyes were replaced by human eyes. These results seem to contradict the idea that face preference reflects an attraction toward human eyes (Baron-Cohen, 1994; Farroni et al., 2005) and seem to contrast previous studies showing that newborns preferred to look at faces with open eyes and with a direct gaze (Batki et al., 2000; Farroni et al., 2002, 2006). However, all these data need to be interpreted with caution because stimuli were never paired as for the low-level variables. Consequently all these preferences might be attributed to a difference in low-level variables such as the difference in spatial frequencies components.

An alternative explanation might be related to the processing of the overall configuration of the face. Possibly, the processing of the eyes might be limited, since newborns might pay more attention to the external parts of faces (Pascalis et al., 1995), especially when eyes are embedded in a non-human primate face with a salient external contour emphasized by fur. However, this explanation is unlikely because newborns attend equally to internal and external features of faces (Turati et al., 2006).

A more convincing explanation would be that newborns process faces holistically and sensitivity for human eyes per se is not inborn but emerges later due to the extensive experience with conspecifics. This idea is supported by recent eye tracker studies in which 3-month-old infants look longer at the eyes of the human face when contrasted with a monkey face (Di Giorgio et al., 2013; Dupierrix et al., 2014). So, it appears that 3 months of exposure to human eyes is sufficient to drive infants’ attention toward the more experienced human eyes (Dupierrix et al., 2014).

Overall, data are in line with the hypothesis that the face-perception system becomes tuned to human faces and human eyes during development as a function of visual experience (Nelson, 2001; Pascalis et al., 2002; Pascalis and Kelly, 2009; Di Giorgio et al., 2013; Dupierrix et al., 2014).

The presence of the perceptual narrowing process with the most experienced faces is supported by eye tracker studies that showed different patterns of exploration for different categories of faces (Liu et al., 2011; Di Giorgio et al., 2013). For instance, the visual scanning paths of 4- to 9-month-old Asian infants presented with same and other-race faces are different as a function of the nature of the stimulus, demonstrating developmental changes in the face processing strategies. For instance, with age, infants tend to look longer at the internal features embedded in the same-race face but not in the other-race faces (Liu et al., 2011).

All together these data corroborate, once more, the idea that newborns’ visual attention is mainly triggered by the low-level perceptual properties of the visual stimuli, whereas, starting from 3 months of life, visual preferences become specific for faces and, specifically, with the more experienced faces, such as human faces or faces that belong to infants’ ethnic group.

From a neural point of view, the perceptual narrowing process consists of a progressive and gradual specialization and localization of the cortical brain areas involved in face processing (Johnson, 2000). Indeed, at birth these circuits respond to a wide range of visual stimuli but later, during development and thanks to visual experience, these cortical circuits became more and more selective to only some categories of visual stimuli, such as experienced face, causing a more localized and specialized neural response. For instance, studies that performed positron emission tomography (PET) scans suggested that, by 2–3 months of age, there are the first signs of cortical specialization for faces (Tzourio-Mazoyer et al., 2002). Moreover, ERPs studies demonstrated that, at a neural level, 6-month-old infants differentiate faces from objects (de Haan and Nelson, 1999) and, interestingly, also human faces from monkey faces (de Haan et al., 2003). Further, near-infrared spectroscopic studies (NIRS) have provided new evidence of cortical regions in the infant brain already devoted to face processing (see Otsuka, 2014, for a review).

Overall, these findings are in line with the idea that the face-perception system is the product of a conjunction of evolutionary inheritance and of an experience-dependent process of learning after birth (de Schonen, 1989; Sai, 2005; Pascalis and Kelly, 2009; Slater et al., 2010) and that the system becomes finely tuned by the visual experience in a specie-specific environment. This specialization corresponds to an improvement in the discrimination of stimuli predominant in the environment and to a decline in the discrimination of stimuli not frequently experienced in the environment. What is currently less understood is the nature of the mechanisms responsible of the perceptual narrowing and of the maintenance or facilitation with experience. One possible neural mechanism that guides perceptual narrowing may be the neural pruning phenomenon (Scott et al., 2007). Indeed, early in life there is an exuberance of synaptic connections in the brain, which are pruned in order to reach adult levels over time. Therefore, it is plausible to hypothesize that the decline in face discrimination ability for certain stimuli coincides with this pruning process.

### How Newborns and Infants Recognize Faces

This part of the paper will discuss how faces are recognized and whether the computations to encode, store and retrieve information are special for faces since birth. From a developmental point of view, it is important to investigate whether infants from birth have the capacity to extract and process both the featural and the configural information present in a face, and how the face processing strategies change and become face-specific as a function of visual experience.

It’s a matter of fact that newborns, despite their immature visual system, are able to recognize individual faces. After the habituation phase with a picture of a female stranger’s face, newborns looked longer at a new face compared to the familiar one, demonstrating their ability to learn a specific individual face to which they are repeatedly exposed (Pascalis and de Schonen, 1994). In addition, the mother’s face is recognized and preferred over a female stranger’s face within hours from birth (Bushnell et al., 1989; Pascalis et al., 1995; Bushnell, 2001; Sai, 2005). Despite this newborns’ learning ability, which is the nature of the operations that occurs on face recognition at birth and in early infancy is still an open question.

Data collected in our lab employing face-like, real faces and geometric stimuli converge to suggest that, at least at birth, the operations involved in face processing are the same that occur to process any visual object. For instance, newborns are able to discriminate between arrays that are identical with respect to the global characteristics (i.e., columns of filled or unfilled elements), but differed as for to the shape of the filled elements contained within the two filled columns (i.e., square elements vs. diamond elements). This result shows that newborns are able to discriminate the individual elements of an array and can organize such elements into a holistic percept (Farroni et al., 2000). The same results have been obtained with face-like patterns since newborns discriminated between schematic face-like that differed exclusively for the shape of the internal local elements (Simion et al., 2002).

Together, these data support the hypothesis that newborns possess a general visual pattern-learning mechanism that enables them to encode, retrieve, and thus recognize as familiar, visual stimuli independently of whether they are faces or not. The learning mechanism responsible of face recognition is not specific for faces but, rather, operates in a similar fashion for all types of visual stimuli (de Schonen and Mancini, 1995; de Schonen et al., 1998; Johnson and de Haan, 2001).

In line with the presence of this general visual pattern-learning mechanism, active both for faces and non-face stimuli, infants from birth are able to perceive and recognize the invariant perceptual characteristics of a wide range of visual stimuli. For instance, newborns are able to perceive objects and faces as invariant across the retinal changes due to modifications in slant or distance (Slater and Morison, 1985; Slater et al., 1990), both when physical (i.e., simple or complex geometrical patterns) and social objects are available in the environment. For instance, it has been demonstrated that newborns are able to process the invariant features of a face regardless of changes in slant relative to the observer (Turati et al., 2008).

Overall, the general visual pattern- learning mechanism seems to operate on non-face-like, face-like configurations and real faces and is thought to be sensitive to those coarse visual cues of a face or non-face stimuli strictly dependent on LSF that convey configural information.

Indeed, evidence demonstrated that the visual information newborns use to process and recognize a face is triggered by low-rather than high-spatial frequencies (de Heering et al., 2007b). Basically, this is due to the fact that, configural information, is processed mainly by the right hemisphere (de Schonen and Mathivet, 1989; Deruelle and de Schonen, 1991, 1998; de Schonen et al., 1993). Deprivation of early visual input to the right hemisphere, due to a bilateral congenital cataract, led to impaired configural processing (Le Grand et al., 2003). Since the right hemisphere matures before and at a faster rate than the left hemisphere, newborns and young infants are sensitive to configural information more than to features in both faces and non-faces (de Schonen and Mathivet, 1990). In effect, the same LSF range is critical in producing the global/local advantage found when newborns process hierarchical stimuli (Macchi Cassia et al., 2002). Employing hierarchical patterns in which larger figures (i.e., cross or rhombus) are constructed from the same set of smaller figures, it has been demonstrated that newborns are able to discriminate both the local and the global levels. However, recognition of the local features was impaired in the condition when information at the global level interfered with identification of the local features (Macchi Cassia et al., 2002). This asymmetrical interference might be used to interpret the inversion effect obtained in the inner features condition with faces. That is, when the face is in the upright orientation newborns encode both levels (i.e. local and global) with a superiority of the global/configural one, which allows recognition of the face. In contrast, when the face is turned upside- down, newborns are impaired to use the global/configural information and, due to the sensitivity to LSF, cannot rely upon the only use of the featural information (Turati et al., 2006). Collectively, findings reported here demonstrated that newborns are sensitive to configural information both to faces and non-faces stimuli due to constraints of their visual system.

However, since in adults configural processing is specific for faces and it has been attributed to the extensive experience with faces during lifetime, from a developmental point of view it seems crucial to investigate when faces start to become special and start to be processed differently from objects (see Hoel and Peykarjou, 2012). Some studies demonstrated that infants start to process differently upright and inverted faces within the first months of life, providing evidence for an early face inversion effect. For instance, Turati et al. (2004) showed that the face inversion affected 4-month-olds’ face recognition abilities. In the same vein, 4-month-old infants’ visual scanning paths are different as a function of the orientation in which the face was presented (Gallay et al., 2006; see also Kato and Konishi, 2013). At a neural level, two ERP components (i.e., N290 and P400) are found to be indicative of a face processing ability in early infancy (de Haan et al., 2002; Halit et al., 2003; Scott and Nelson, 2006; Scott et al., 2006). ERPs studies conducted with 6-month-old infants revealed that the P400, a precursor of the adult N170, was modulated by inversion already at this age: inverted faces demonstrated greater amplitude negativity than upright faces (Webb and Nelson, 2001; de Haan et al., 2002). Interestingly, although there are no behavioral studies that directly compare inversion effect for faces vs. objects in infants, a recent NIRS study demonstrated that inversion effect for faces and objects differently modulates brain activation in 5- and 8-month-old infants (Otsuka et al., 2007). Further studies demonstrated that, starting in early childhood, the stimulus inversion affects disproportionately faces compared to objects (Picozzi et al., 2009), corroborating previous results with older children (Carey and Diamond, 1977; Teunisse and de Gelder, 2003).

As for the composite face effect, a recent study reported, for the first time, that 3-month-old infants, as well as adults, process faces holistically. Specifically, infants have shown to be more accurate in recognizing the familiar top-half of a face in the misaligned condition as compared to the aligned condition (Turati et al., 2010). Interestingly, although both adults and infants showed the composite face effect, their performance differed in the misaligned condition. In effect, adults looked longer at the novel top half, whereas infants looked longer at the familiar top half. This result demonstrates that the tuning toward configural information appears very early in life, but experience progressively refines early configural strategies in face processing. Employing the same composite face paradigm and extending previous findings (Carey and Diamond, 1994; Mondloch et al., 2007), some studies demonstrated that holistic face processing is fully mature at 4 years of age (de Heering et al., 2007a) and is selective for faces at 3.5 years of age (Macchi Cassia et al., 2009).

Intriguingly, all the studies reported here confirm that visual experience is critical for the typical development of face processing. However, at present how early visual experience shapes the neural mechanisms underlying face processing is not well understood. In light of this, future studies should be conducted to better understand what kind of visual experience is more effective to render the face processing system specialized and the sensitive periods during development (see Scott et al., 2007). A more recent ERP study conducted with infants from 6 to 9 months has attempted to answer this question.

In this study, a neural specialization indexed by a different modulation of P400 for upright compared to inverted monkey faces, was found in infants who have received a training of 3 months with monkey faces labeled at the individual-level (i.e., a single monkey face associated with a name). Infants in this group showed an inversion effect for monkey faces. In contrast, no effects were found in infants who received a training with the same monkey faces labeled at the categorical-level (i.e., “monkey” as the name for all faces presented), demonstrating that the different experiences (i.e., categorical vs. individual learning experiences) affected in a different way face processing and neural specialization for faces during development (Scott and Monesson, 2010).

Taken together, the studies reviewed here demonstrated that at birth, due to the presence of certain constraints of the visual system (e.g., sensitivity to LSF), newborns apply the same strategies to recognize and process both faces and non-faces similarly, corroborating the idea of the existence of a general visual pattern-learning mechanism. Then, during development, thanks to the specific visual experience with certain kind of stimuli, the system becomes specialized to process differently objects and social stimuli.

## Conclusion

Overall, the studies carried out with newborns demonstrated the presence, since birth, of pre-wired domain relevant attentional biases toward faces and the role of experience in shaping the face processing system.

As for face detection, here we suggest that faces are not special visual stimuli for newborns and that a specific face-sensitive mechanism is not required to explain face preference since birth. The reviewed evidence speaks in favor of the hypothesis that faces might be preferred at birth because they are a collection of preferred structural (i.e., up-down asymmetry, congruency, etc.) and configural properties that other stimuli may also possess. Consequently, the debate is still open and further studies need to be carried out to disentangle the question about general or specific biases underlying face preference at birth. Further, it seems relevant to investigate whether the activation of the subcortical route in newborns and in adults, putatively active throughout the lifespan (Tomalski et al., 2009), is elicited or not by the same visual stimuli during development and the nature of the interaction between the cortical and subcortical routes in face processing along lifespan.

In addition, future studies are needed on the nature of face representation at birth because we are far from a conclusive answer about the best stimulus that elicits face preference at birth. Some controversial studies about the effect of contrast polarity (Farroni et al., 2005) and the role of the eyes in triggering face preference at birth (see Dupierrix et al., 2014) suggest to further investigate, both with behavioral and neuroimaging studies, what low-level visual cues, such as the high contrast area of the human eyes and the pupil, may render them so important in the first months of life and whether their relevance changes over time.

Furthermore, future studies should investigate what is the nature of the mechanisms responsible of the perceptual narrowing process that occurs during development and, even more important, what is the visual experience that is more effective to guide the specialization of the system to process faces during the sensitive and/or critical periods during development. In particular, electrophysiological studies are needed to investigate how the infant brain works during development in response to faces.

In the same vein, how and when faces become special stimuli and start to be processed differently from objects are intriguing open questions. Future studies should directly compare visual processing strategies employed for faces and for objects by using the same paradigms at different time points during development in order to track a developmental trajectory of the face processing specialization.

One of the main purpose that guides such research should be to increase the knowledge about the typical developmental trajectories in order to identify infants who deviate from them (i.e., infants at high-risk for autism) and to promote screening and intervention programs when the brain is more plastic and receptive to changes.

Overall, the evidence is consistent in demonstrating a progressive functional and neural specialization of the face-system. The data reviewed here speak in favor of the idea that, in order to develop in its adult-like expert form, the face-system may not require a highly specific input (i.e., a face-specific bias). Rather, it is plausible to hypothesize that the presence of some domain-relevant attentional biases at birth is sufficient to set up and to drive the system toward the gradual and progressive structural and functional specialization that emerges later during the development thanks to the visual experience that infants have in their species-specific environment.

### Conflict of Interest Statement

The authors declare that the research was conducted in the absence of any commercial or financial relationships that could be construed as a potential conflict of interest.
